# Archives of Electrology and Neurology

**Published:** 1874-08

**Authors:** 


*Archives of Electrology and Neurology: A Journal of Electro-Therapeutics and Nervous Diseases.* Edited by GEO. M. BEARD, A. M., M. D. Vol. I, No. 1.

We welcome this new Journal to our list of exchanges; devoted to a subject which is daily attracting more attention from physicians it should receive the hearty support of all who are desirous of the advancement of scientific medicine. The editor, Dr. Beard, stands high as an observor in this special branch of medicine, and under his supervision we may expect the Journal will fulfill the promises which its first number makes.

